# The impact of data quality monitoring of a multicenter prospective registry of cardiac implantable electronic devices

**DOI:** 10.1016/j.mex.2023.102454

**Published:** 2023-10-20

**Authors:** Sarah Caroline Martins Saucedo, Katia Regina Silva, Laísa de Arruda Silva, Jéssica Moretto Crivelari, Roberto Costa

**Affiliations:** Unidade de Estimulação Elétrica e Marcapasso, Instituto do Coração do Hospital das Clínicas da Faculdade de Medicina da Universidade de São Paulo (InCor-HCFMUSP), Sao Paulo, Brazil

**Keywords:** *Data quality monitoring of multicenter registries*, Pacemaker, Artificial, Database, Registries, Data quality, Data management, Data audit, REDCap

## Abstract

Data quality monitoring plays a crucial role in multicenter prospective registries. By maintaining high data accuracy, completeness, and consistency, researchers can improve the overall quality and reliability of the registry data, enabling meaningful conclusions and supporting evidence-based decisions. The purpose of the present study was to evaluate data quality metrics (completeness, accuracy, and temporal plausibility) of a Multicenter Registry of Cardiac Implantable Electronic Devices (CIEDs) and to perform a direct data audit of a random sample of records to assess the agreement levels with the source documents. The CIED Registry was a prospective, multicenter, real-world observational study carried out from January 2020 to December 2022 in five designated centers across Sao Paulo, Brazil. We assessed the data quality of the CIED Registry by using two distinct approaches:

• Dynamic data monitoring using features of the REDCap (Research Electronic Data Capture) software, including data reports and data quality rules
• Direct data audit in which information from a random sample of 10 % of cases from the coordinating center was compared with original source documents

Our findings suggest that the methodological approach applied to the CIED Registry resulted in high data completeness, accuracy, temporal plausibility, and excellent agreement levels with the source documents.

## Graphical abstract

**Figure:**
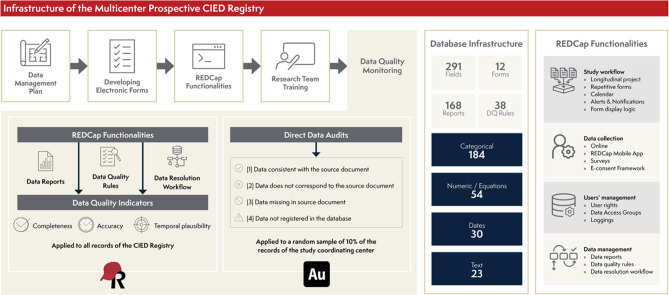
Infrastructure of the Multicenter Prospective CIED Registry.

Specifications table

**Table:** 

Subject area:	Medicine and Dentistry
More specific subject area:	*Cardiac Device Registry*
Name of your method:	*Data quality monitoring of multicenter registries*
Name and reference of original method:	*NA*
Resource availability:	*NA*

## Method details

### Context and significance

Prospective registries with real-world data are a valuable source of clinical evidence that is of significant importance in decision-making, improving the quality of healthcare, formulating evidence-based healthcare policy, and patient safety surveillance [1]. Despite the large volume of publications on clinical registries in the field of Cardiology [2,3], registries of cardiac implantable electronic devices (CIEDs) such as pacemakers, implantable cardioverter-defibrillators (ICDs) and cardiac resynchronization therapy (CRT) are still uncommon in the scientific literature [4,5].

There are many challenges in conducting prospective multicenter registries. The main thing is to have a single database for all centers which allows access to different user profiles in a secure way and enables monitoring data quality in real time [6,7]. In this sense, adopting digital tools for electronic data collection and management can help overcome these logistical and technological challenges, bringing greater efficiency to all the study life cycle [8].

Evaluating data quality metrics from prospective registries has gained great prominence in the literature in recent years [9,10]. Although there is no consensus on the main metrics which should be adopted, the most cited indicators include completeness, accuracy, and temporal plausibility [11], [12], [13]. At the same time, direct data auditing, which involves comparing collected data with primary information sources, has been adopted as an additional strategy to ensure the quality of prospective clinical registries [14,15].

The hypothesis of the present study is that adopting strategies aimed at monitoring data quality can minimize the error rates and inconsistencies inherent in prospective multicenter registries. Thus, based on implementing infrastructure for data collection and management applied to a Multicenter and Prospective CIED Registry, the purpose of the present study was to evaluate the most common data quality metrics (completeness, accuracy, temporal plausibility) and to perform a direct data audit of a random sample of records to assess the agreement levels with the source documents.

### Study design

This is a methodological study designed to evaluate data quality metrics from a Prospective Multicenter CIED Registry.

### Prospective multicenter registry of cardiac implantable electronic devices

The CIED Registry was a prospective, multicenter, real-world observational study carried out from January 2020 to December 2022 in five designated centers across the state of Sao Paulo, Brazil. The study was coordinated by a referral center for Cardiac Pacing and Electrophysiology located in the city of Sao Paulo, Brazil. All individuals who underwent initial CIED implantation or reoperations for maintenance and/or treatment of complications related to the cardiac device were consecutively included in the CIED Registry.

Data derived from electronic health records and hospital administrative systems were collected at three different times: index hospital admission, at 30 and 180 days after discharge. Demographic, clinical, surgical, and discharge data were also collected at hospital admission. In addition to collecting clinical follow-up data that evaluated complications and readmissions which occurred in the postoperative phase (30 and 180 days after discharge), patient-reported outcome (PRO) measures were also obtained using standardized instruments in a convenience sample.

### Infrastructure of the prospective multicenter registry of cardiac implantable electronic devices

The data collection and management infrastructure of the CIED Registry included the following steps: (1) Data management plan; (2) Defining data element terminology; (3) Developing electronic case report forms (e-CRF) using REDCap (Research Electronic Data Capture); (4) Customization of specific functionalities of REDCap; (5) Research team training; (6) Dynamic monitoring of data quality with REDCap functionalities; and (7) Direct data auditing [16].

Developing e-CRF in REDCap requires careful planning and adherence to good practices to ensure the overall quality of the research protocol. In this study, the format and terminology of required data elements were defined in accordance with international standards, as previously published [16], comprising a comprehensive data dictionary that encompassed all data points to be collected during the patient's journey. Related data elements were logically grouped into specific forms (demographic, clinical, surgical, discharge, follow-up) to facilitate efficient data entry. The use of appropriate field types (text, drop-down list, radio buttons, and checkboxes) was crucial to optimize data entry, and most importantly, to ensure that exported data formats met the requirements of downstream analyses. Additional resources, such as numeric field validation, branching logics and automatic calculated fields were adopted in several database forms, aiming to minimize potential typing errors and facilitate data quality monitoring.

The database structure consisted of 12 electronic forms, making a total of 291 variables, as follows: 184 categorical variables; 54 numeric, among which 50 resulted from automatic calculations; 30 variables structured in date format and 23 variables that allowed the use of free text. Additional details regarding the database structure are presented in Supplementary Material (Table S1).

The main REDCap functionalities used in the study were longitudinal events, offline data collection through the REDCap Mobile App, user access control, creation of multicenter groups, the calendar for scheduling patient evaluations and the electronic consent form framework. An extensive list of REDCap features used in this project can be found in Supplementary Material (Table S2).

The research team was trained according to the attributes of each user profile. The contents taught during the training sessions included: overview of the study scope, electronic data collection, monitoring the study workflow, correcting data inconsistencies, and monitoring data quality. These training sessions were conducted by videoconferences associated with the sending of tutorials.

### Data collection

Data entry personnel included research coordinators, nurses, physicians, and physicians-in-training (residents) who voluntarily agreed to participate as academic partners. All data, except the PRO measures, were abstracted manually from the patient's electronic medical records to the REDCap database. Only in the coordinating center, surgical data were obtained using the REDCap Mobile App in the operating room by the physicians-in-training; after the procedure, however, surgical data was also registered in the patient's chart.

REDCap surveys were used to capture self-reported PRO measures at follow-up time points. The survey link was sent to the participants via email, SMS, or WhatsApp message, depending on the participant's preference.

### Monitoring data quality

The study coordinating center monitored information from the prospective multicenter CIED registry using a systematic approach based on a weekly basis to ensure data quality. This monitoring included creating data reports and specific data quality rules, being applied to all datasets. For this step, the main data quality metrics which could be evaluated using REDCap's features were chosen, including completeness, which refers to the amount of complete data; accuracy, which refers to the absence of inconsistencies; and temporal plausibility, which comprises the amount of data inserted within the times pre-established by the study [17].

### Direct data audit

Direct data auditing was carried out to assess the veracity of the information entered in the database, being applied to a random sample of 10 % of the records of the study coordinating center, selected through the R Studio software. Data from the prospective multicenter CIED Registry were compared with the original source documents using forms developed in REDCap to classify each of the audited variables into the following options: (1) the data corresponds to the source document; (2) the data does not correspond to the source document; (3) the data does not exist in the source document; and (4) the data was not registered in the database, in accordance with international recommendations [18].

### Studied variables

Data quality monitoring was applied to all database variables, including demographic data (age, sex and race), clinical data (baseline heart disease, functional class of heart failure and associated comorbidities), surgical data (type of procedure, type of CIED, intraoperative complications), hospitalization data (need for daily stays in the intensive care unit, hospital complications and final outcome), data from the 30 and 180-day clinical follow-up (postoperative complications, readmissions and reoperations in the period), the standardized instruments of PRO measures and the study closure.

### Statistical analysis

The quality metrics of each of the studied variables were analyzed descriptively, calculating absolute and relative frequencies, as well as central tendency and dispersion measures. The overall rate of each of the data quality metrics was calculated along with their respective confidence intervals (CI) to complement the descriptive analysis.

The Kappa coefficient (k) was used for categorical variables and the intraclass correlation coefficient (ICC) for continuous variables to estimate the agreement between the data sample from the Registry that were collected before and after performing the data audit stage. The significance level used for the tests was 5 %.

## Method validation

### Participants of the CIED registry

During the study period, a total of 2631 consecutive patients were included in the CIED Registry and were followed up for a period of 6.1 ± 1.3 months. Thus, the data quality metrics presented in the next section refer to a total of 2631 records.

### Data quality metrics

The overall rate of the studied data quality metrics showed satisfactory results with averages of 99.9, 99.8, and 96.3 % for completeness, accuracy, and temporal plausibility, respectively. The completeness and accuracy analysis showed similar behavior for the different datasets evaluated. Regarding completeness, the demographic, clinical, surgical and outcome variables reported by patients who presented 100 % complete data stood out (Table 1). Data quality metrics were also evaluated according to the research site, and the results demonstrated the same pattern observed in global rates as presented in Supplementary Material (Table S3).

**Table 1:** Data completeness and accuracy rate of the multicenter prospective CIED registry.

Variables	Completeness (%)	Accuracy (%)
**Demographic data**		
Date of birth	100	100
Date of hospital admission	100	100
Age	100	100
Sex	100	100
Race	100	–
**Clinical data**		
Heart disease	100	97.9
Heart failure functional class (New York Heart Association)	100	100
Hypertension	100	–
Prior acute myocardial infarction/coronary artery disease	100	99.8
Heart failure	100	100
Atrial Fibrillation/Atrial Flutter	100	100
Cardiac arrest/unstable ventricular tachycardia	100	99.8
Previous heart transplant	100	–
Valvular heart disease/valve prostheses	100	–
Prior endocarditis	100	–
Diabetes mellitus	100	–
Chronic kidney failure	100	–
Hemodialysis	100	–
Previous stroke/transient ischemic attack	100	–
Chronic obstructive pulmonary disease	100	–
Recent neoplasm	100	–
Use of oral anticoagulant	100	98.4
**Procedure information**		
Procedure date	100	100
Type of procedure	100	100
Type of anesthesia	100	100
Indication for initial CIED implant	100	100
Indication for CIED reoperation	100	100
Main procedure performed	100	100
Cardiac device at the end of the procedure	100	100
Intraoperative death	100	100
Intraoperative complications	100	100
Cardiopulmonary arrest	100	100
Unstable cardiac arrhythmia	100	100
Respiratory failure	100	100
Cardiac tamponade	100	100
Avulsion of cardiac structures / Venous laceration	100	100
Unstable bleeding	100	100
Coronary sinus injury	100	100
Valve lesion	100	100
Hemothorax requiring drainage	100	100
Minimal hemothorax with no need for drainage	100	100
Pneumothorax requiring drainage	100	100
Minimal pneumothorax with no need for drainage	100	100
Pericardial effusion requiring drainage	100	100
Minimal pericardial effusion with no need for drainage	100	100
Bleeding without hemodynamic instability	100	100
Lead dislodgement	100	100
Migration of lead fragments	100	100
Allergic reaction	100	100
**Hospital discharge data**		
Need for daily stays in the intensive care unit	99.9	100
Hospital discharge date	99.9	100
Outcome of hospitalization	99.9	100
Length of hospital stay (days)	99.9	100
Reason for extended hospital stay time	95.6	100
Surgical or medical complications during hospitalization	99.9	100
Cardiopulmonary arrest	100	100
Unstable cardiac arrhythmia	100	100
Cardiac tamponade	100	100
Unstable bleeding	100	100
Hemothorax requiring drainage	100	100
Pneumothorax requiring drainage	100	100
Hematoma requiring surgical approach	100	100
Pericardial effusion	100	100
Lead dislodgement	100	100
Pulmonary embolism	100	100
Atrial fibrillation	100	100
Acute myocardial infarction	100	100
Stroke	100	100
Sepsis	100	100
**Data from the 30-day clinical follow-up**		
Date of visit/contact	99.9	–
Pocket infection	99.9	–
Cardiac device related infection	99.9	–
Infection not related to the cardiac device	99.9	–
Lead dysfunction	99.9	–
Lead dislodgement	99.9	–
Venous thrombosis in ipsilateral upper extremity	99.9	–
Pulmonary embolism	99.9	–
Hospital readmission	99.9	–
Date of hospital readmission	100	–
Need to perform a new surgical procedure	99.9	–
Date of the new surgical procedure	100	–
**Data from the 180-day clinical follow-up**		
Date of visit/contact	99.9	–
Pocket infection	99.9	–
Cardiac device related infection	99.9	–
Infection not related to the cardiac device	99.9	–
Lead dysfunction	99.9	–
Lead dislodgement	99.9	–
Venous thrombosis in ipsilateral upper extremity	99.9	–
Pulmonary embolism	99.9	–
Hospital readmission	99.9	–
Date of hospital readmission	100	–
Need to perform a new surgical procedure	99.9	–
Date of the new surgical procedure	100	–
**Questionnaires applied in the 30-day follow-up**		
EQ-5D-3L	100	–
Florida Patient Acceptance Scale (FPAS)	100	–
Florida Shock Anxiety Scale (FSAS)	100	–
Hospital Consumer Assessment of Healthcare Providers and System (HCAHPS)	100	–
**Questionnaires applied in the 180-day follow-up**		
EQ-5D-3L	100	–
Florida Patient Acceptance Scale (FPAS)	100	–
Florida Shock Anxiety Scale (FSAS)	100	–
**Study closure data**		
Date of last visit/contact	99.9	100
Outcome	99.9	100
Death during the study period	99.9	100
Date of death	100	100
Cause of death	100	98.7

The temporal plausibility rate was lower in the variables related to the study closure (90.8 %) and the clinical follow-up of 180 days (94.5 %). On the other hand, the data quality metrics related to the variables of the standardized PRO questionnaires had the best performance (98.8–99.9 %), with practically all cases inserted within the times pre-established by the study (Table 2).

**Table 2:** Temporal plausibility rate of data from the multicenter prospective CIED registry.

Variables	Temporal plausibility (%)
**Data from the 30-day clinical follow-up**	
Date of visit/contact	97.3
Pocket infection	97.3
Cardiac device related infection	97.3
Infection not related to the cardiac device	97.3
Lead dysfunction	97.3
Lead dislodgement	97.3
Venous thrombosis in ipsilateral upper extremity	97.3
Pulmonary embolism	97.3
Hospital readmission	97.3
Date of hospital readmission	98.0
Need to perform a new surgical procedure	97.3
Date of the new surgical procedure	96.0
**Data from the 180-day clinical follow-up**	
Date of visit/contact	94.5
Pocket infection	94.5
Cardiac device related infection	94.5
Infection not related to the cardiac device	94.5
Lead dysfunction	94.5
Lead dislodgement	94.5
Venous thrombosis in ipsilateral upper extremity	94.5
Pulmonary embolism	94.5
Hospital readmission	94.5
Date of hospital readmission	98.9
Need to perform a new surgical procedure	94.5
Date of the new surgical procedure	96.3
**Questionnaires applied in the 30-day follow-up**	
EQ-5D-3L	99.7
Florida Patient Acceptance Scale (FPAS)	99.6
Florida Shock Anxiety Scale (FSAS)	98.8
Hospital Consumer Assessment of Healthcare Providers and System (HCAHPS)	99.7
**Questionnaires applied in the 180-day follow-up**	
EQ-5D-3L	99.9
Florida Patient Acceptance Scale (FPAS)	99.9
Florida Shock Anxiety Scale (FSAS)	99.4
**Study closure data**	
Date of last visit/contact	90.8
Outcome	90.8
Death during the study period	90.8

### Direct data auditing

A direct data audit was performed on 200 (10.1 %) random records from the study coordinating center. A total of 125 (0.9 %) errors were found among the 13,800 audited variables. The agreement rate between the records and the information present in the source documents ranged from 97.8 to 99.6 %, with data related to the surgical procedure presenting the best rates (Table 3).

**Table 3:** Findings from the direct data audit performed on a sample of 200 cases from the Multicenter Prospective CIED Registry.

Data audit findings	Demographic data	Clinical data	Procedure data	Discharge data
Classification of findings				
Data consistent with the source document	99.3 %	97.8 %	99.6 %	99.4 %
Data does not correspond to the source document	0.5 %	1.7 %	0.4 %	0.2 %
Data missing in source document	0.1 %	0.1 %	0 %	0 %
Data not registered in the database	0.1 %	0.4 %	0 %	0.4 %
Overall disagreement rate	0.7 %	2.2 %	0.4 %	0.6 %
Total number of variables audited	1000	3400	5200	4200
Total number of errors	7	74	19	25

The agreement analysis between the data from the registry that were collected before and after the direct data audit stage showed that both categorical and numerical variables showed excellent levels of agreement (*k* > 0.90 and ICC = 1.00) (Table 4). The mean Kappa coefficient was 0.939 ± 0.07, ranging from 0.660 to 1.000. The variables with the lowest reliability coefficients were presence of endocarditis prior to the surgical procedure (*k* = 0.660, 95 % CI 0.301 - 1.000, *P*<0.001), heart failure functional class (*k* = 0.818, 95 % CI 0.746 - 0.889, *P*<0.001) and reason for the initial CIED implant (*k* = 0.833, 95 % CI 0.735 - 0.930, *P*<0.001). Both age and length of hospital stay correlated perfectly with data from source documents (ICC = 1.000, *P*<0.001).

**Table 4:** Analysis of agreement between data collected in the Multicenter Prospective CIED Registry and the results of the direct data audit.

Variables	ICC	Kappa	SE	95 % CI	P
**Demographic data**					
Age	1.000	–	–	1.000 - 1.000	<0.001
Sex	–	1.000	0.000	1.000 - 1.000	<0.001
Race	–	0.954	0.032	0.891 - 1.000	<0.001
**Clinical data**					
Heart disease	–	0.918	0.023	0.872 - 0.963	<0.001
Heart failure functional class (New York Heart Association)	–	0.818	0.037	0.746 - 0.889	<0.001
Hypertension	–	0.949	0.023	0.904 - 0.993	<0.001
Prior Acute Myocardial Infarction/ Coronary Artery Disease	–	0.895	0.046	0.804 - 0.986	<0.001
Heart failure	–	0.918	0.028	0.862 - 0.974	<0.001
Atrial Fibrillation/Atrial Flutter	–	0.973	0.019	0.935 - 1.000	<0.001
Cardiac arrest/unstable ventricular tachycardia	–	0.954	0.032	0.891 - 1.000	<0.001
Valvular heart disease/valve prostheses	–	0.984	0.016	0.953 - 1.000	<0.001
Prior endocarditis	–	0.660	0.183	0.301 - 1.000	<0.001
Diabetes mellitus	–	0.945	0.027	0.892 - 0.998	<0.001
Chronic Kidney Failure	–	0.973	0.027	0.920 - 1.000	<0.001
Hemodialysis	–	1.000	0.000	1.000 - 1.000	<0.001
Previous stroke/transient ischemic attack	–	0.965	0.035	0.897 - 1.000	<0.001
Chronic obstructive pulmonary disease	–	1.000	0.000	1.000 - 1.000	<0.001
Recent neoplasm	–	1.000	0.000	1.000 - 1.000	<0.001
Use of oral anticoagulants	–	0.961	0.023	0.917 - 1.000	<0.001
**Procedure information**					
Type of procedure	–	1.000	0.000	1.000 - 1.000	<0.001
Reason for procedure: initial implants	–	0.833	0.050	0.735 - 0.930	<0.001
Reason for the procedure: reoperations	–	0.885	0.044	0.799 - 0.970	<0.001
Main procedure performed	–	0.992	0.008	0.977 - 1.000	<0.001
Cardiac device at the end of the procedure	–	0.982	0.012	0.958 - 1.000	<0.001
Need for daily stays in the intensive care unit	–	0.982	0.018	0.946 - 1.000	<0.001
Outcome	–	1.000	0.000	1.000 - 1.000	<0.001
Length of hospital stay (days)	1.000	–	–	1.000 - 1.000	<0.001
Reason for extended hospital stay time	–	0.901	0.042	0.818 - 0.984	<0.001
Surgical or medical complications during hospitalization	–	0.921	0.079	0.766 - 1.000	<0.001

ICC= intraclass correlation coefficient; CI= confidence interval; SE = standard error.

### Study implications

The importance of multicentric prospective studies with real world data is already well established in the literature and its application has been increasingly widespread in different clinical settings [19]. However, the benefits of this type of study are intrinsically related to the availability of a database infrastructure combined with continuous monitoring of the quality of information [20]. Although these strategies are essential, they are often placed in the background, compromising the reliability and reproducibility of the results of scientific research [[21], [22]]. Thus, the present study aimed to evaluate the impact of adopting strategies aimed at monitoring the data quality applied to a prospective multicenter registry of cardiac devices.

The CIED Registry covered all types of cardiac devices, as well as any category of surgical procedure related to cardiac pacing. In addition to care quality indicators, this record included an evaluation of PROs and the patient's experience with hospital care received during hospitalization through standardized questionnaires.

Adopting the REDCap as the software of choice for the present study enabled implementing strategic actions for managing and monitoring the data quality, which is crucial to minimize error rates and inconsistencies that may occur in the data collection stage [[22], [23]]. When used in a systematic way, these actions enable identifying and measuring missing data rates, outliers and inconsistencies that are the main metrics of data quality [9].

Measuring data quality metrics has gained great prominence in publications of observational studies with real-world data and clinical trials [23,24]. Although there is no consensus as to the main metrics that should be adopted, the most cited metrics in the literature include completeness, accuracy, and temporal plausibility [11], [12], [13]. Completeness is the most used because it is easy to assess and one of the most important criteria to assess data quality, considering that the absence of information makes the analysis of other indicators unfeasible [17]. The classic rule is that the missing data rate should not exceed values between 5 and 10 % [25]. The present study obtained an average rate of 0.1 % of missing data, which practically eliminated the difficulties in conducting data analysis provided for in the research protocol.

Accuracy is the second most adopted metric in the literature, mainly for measuring data validity [11]. It is generally expected that the accuracy rate of research data is as close as possible to 100 %; however, most studies have reported values ranging from 97 to 99 % [11,26]. The average accuracy in the experience of the CIED Registry was very close to 100 %, probably due to the fact that continuous inconsistencies monitoring strategies were adopted.

The temporal plausibility measures the punctuality of data collection in relation to the predetermined times for the study; therefore, it is generally restricted to prospective designs [12]. This was the data quality metric that presented the lowest index in the CIED Registry in relation to the others assessed, ranging from 90.8 % to 99.7 % for data from the study closure and the EQ-5D-3 L quality of life questionnaire, respectively. In this regard, it is worth considering the impact of COVID-19, which made it difficult for patients to attend return appointments within the deadlines that were foreseen for outpatient care. Furthermore, the values of this indicator varied between research sites (Table S3), consequently, the average values may have been influenced by the low values found in two hospitals (45.8 % and 88.5 %, for site 4 and site 1, respectively).

The direct data audit carried out in the present study identified a total of 125 errors in 13,800 audited variables. Although there is no consensus regarding the acceptable error rate in a database, a literature review involving 42 observational studies reported an average rate of 976 errors per 10,000 variables [27]. In parallel with the data audit, an agreement analysis was performed between the data sampling from the CIED Registry that were collected before and after conducting the data audit stage, showing that both the categorical and numerical variables showed excellent agreement levels between the data from the CIED Registry and the source documents, and being higher than the values reported in similar studies [14,15].

Despite being a multicenter study, the coordinating center was responsible for 75.3 % of the total sample, probably because it is a reference center for cardiovascular care and because it receives patients referred from other institutions to perform more complex surgical procedures. In addition, as expected, the COVID-19 pandemic imposed some difficulties on conducting the study. Above all, the sites had a considerable delay in sending the documentation to their respective Research Ethics Committees, and there was temporary suspension of elective surgical procedures in some cases. Thus, the main limitation regarding the study sample was not the total number of registered cases, but rather the impossibility of guaranteeing that all patients operated on in the co-participating sites were included in the CIED Registry.

Although a considerable effort was devoted to performing the direct data audit, it was not possible to apply this step to the sites, mainly due to the Brazilian General Data Protection Law (LGPD) implying specific restrictions for access to the patient's medical record by professionals not directly related to care activities [28].

Our findings suggest that the methodological approach applied to the CIED Registry resulted in high data completeness, accuracy, temporal plausibility, and excellent agreement levels with the source documents. Thus, it is feasible to suggest that data from the prospective multicenter CIED Registry meet the minimum data quality requirements, constituting a reliable, robust, and valid source of information. In addition, the data management infrastructure adopted in this study can be transposable to other medical specialties, improving the overall quality and reliability of patient registries.

## Ethics statements

The study was approved by the Research Ethics Committee of the institution under protocol number 3.799.031. All the procedures in this study were in accordance with the 1975 Helsinki Declaration, updated in 2013. Written informed consent was obtained from all participants included in the study. Data were analyzed anonymously. To guarantee data protection and privacy, this study was carried out in compliance with the Brazilian General Data Protection Law (LGPD) [28].

## Funding sources

The Sao Paulo Research Foundation, Sao Paulo, Brazil (FAPESP), Grant number: 2019/09155–3.

Coordenação de Aperfeiçoamento de Pessoal de Nível Superior-Brasil (CAPES), Grant number: Finance Code001; Master Scholarship 88,887.677970/2022–00.

The funders had no role in the study design, data collection and analysis, decision to publish, or preparation of the manuscript.

## CRediT authorship contribution statement

**Sarah Caroline Martins Saucedo:** Methodology, Data curation, Writing – review & editing. **Katia Regina Silva:** Conceptualization, Methodology, Software, Funding acquisition, Writing – original draft, Writing – review & editing. **Laísa de Arruda Silva:** Data curation. **Jéssica Moretto Crivelari:** Software. **Roberto Costa:** Methodology, Writing – review & editing.

## Declaration of Competing Interest

The authors declare that they have no known competing financial interests or personal relationships that could have appeared to influence the work reported in this paper.

## Data Availability

Data will be made available on request.

## Appendix Supplementary materials

Image, application 1

Image, application 2

Image, application 3
