# Supplementary material for: The impact of data quality monitoring of a multicenter prospective registry of cardiac implantable electronic devices
Source: MethodsX. 2023 Oct 20;11:102454. doi: 10.1016/j.mex.2023.102454 (PMC10618759; doi:10.1016/j.mex.2023.102454)
Supplement: Supplementary file 1 [file mmc1.pdf]

Table S1 - Database structure of the Multicenter Prospective Registry of Cardiac Implantable Electronic Devices.

Data collection forms	Purpose	Variables
Demographic data	Characterization of the patient's demographic profile	Date of birth<br>Date of hospital admission<br>Age<br>Sex<br>Race
Clinical data	Characterization of the patient's clinical profile	Heart disease<br>Heart failure functional class (New York Heart Association)<br>Comorbidities<br>Use of oral anticoagulant
Procedure data	Characterization of surgical procedures, success rate and complications	Procedure date<br>Type of procedure (initial implants or reoperations)<br>Reason for the procedure<br>Main procedure performed<br>Cardiac device at the end of the procedure<br>Intraoperative death<br>Intraoperative complications
Hospital discharge data	Characterization of the hospital journey, complications, and hospital outcomes	Need for daily stays in the intensive care unit<br>Hospitalization outcome (discharge, transfer, death)<br>Length of hospital stay (days)<br>Reason for extended hospital stay time<br>Surgical or medical complications during hospitalization
Data clinical follow-up	Follow-up of clinical evolution 30 and 180 days after hospital discharge	Date of visit/contact<br>Clinical follow-up complications<br>Hospital readmissions<br>Date and reason for hospital readmissions<br>Need to perform a new surgical procedure<br>Date and reason for the surgical procedure
Patient-reported outcomes measures	Outcome measures from the patient's perspective (quality of life, acceptance of the cardiac device, anxiety, and hospitalization experience)	EQ-5D-3L<br>Florida Patient Acceptance Scale (FPAS)<br>Florida Shock Anxiety Scale (FSAS)<br>Hospital Consumer Assessment of Healthcare Providers and System (HCAHPS)
Study closure data	Outcome at the study closure, clinical status, mortality	Date of last visit or phone contact<br>Study outcomes<br>Death during the study period<br>Date of death and cause of death
