# Supplementary material for: The impact of data quality monitoring of a multicenter prospective registry of cardiac implantable electronic devices
Source: MethodsX. 2023 Oct 20;11:102454. doi: 10.1016/j.mex.2023.102454 (PMC10618759; doi:10.1016/j.mex.2023.102454)
Supplement: Supplementary file 2 [file mmc2.pdf]

Table S2 - REDCap functionalities used in the Multicenter Prospective Registry of Cardiac Implantable Electronic Devices.

REDCap functionalities	Details of the function and applicability in the study
Study workflow	
Longitudinal design	Allowed the use of the same form more than once according to the foreseen events; three longitudinal events were defined in the prospective multicenter CIED Registry (Index admission, 30- and 180-day follow-up).
Repetitive forms and/or events	Enabled the use of forms and/or events as many times as necessary. Important function for the study due to the possibility of some patients undergoing more than one surgical procedure during the period.
Calendar	Useful tool for scheduling survey participant assessments. This function considered the time intervals between events that were defined during project configuration. A total of 1,982 appointments were made in the study.
Alerts and Notifications	Sending automatic e-mail alerts with notification of any necessary action that was detected through conditional logic. This additional resource ensured that the data collection of the clinical follow-up phase was carried out within the deadlines foreseen by the study in the sites.
Data collection	
Online	Data collection performed directly in the REDCap software interface, requiring an internet connection and validation of user credentials.
REDCap Mobile App	Offline data collection through an application installed on tablets designated exclusively for research activities. After completing the day's procedures, the data were sent to the server, being automatically synchronized with the database.
Surveys	Allowed the form to be answered by the participant through a link sent by email or text messages (WhatsApp).
e-consent framework	Framework for obtaining the participant's electronic consent. After signing, a PDF file of the document was obtained automatically, which could be made available to participants, in addition to being automatically stored in the database.
User management	
User rights	Management of user access levels to REDCap resources and project forms, enabling customization of different profiles according to the study characteristics.
Data Access Groups (DAGs)	Specific function for multicentric studies which enabled segmenting data and users according to the research center, however maintaining a single and centralized database; five distinct access groups were created for the Prospective Multicenter CIED Registry.
Logging	Enabled tracking of all actions performed by users in the project.

Data quality monitoring	
Data reports	It enabled visualizing data (total or with filtering) in table format or graphs with descriptive statistics, as well as export to different statistical software. In all, 152 reports were prepared to detect missing data and inconsistencies, in addition to monitoring outcomes and adverse events in real time.
Data quality syntaxes	A tool which enabled constructing syntaxes to assess inconsistencies in the data, generally by crossing variables that do not match each other well and reflect in some type of discrepancy. In total, 36 data quality syntaxes were created in the Prospective Multicenter CIED Registry.
Data resolution workflow	Workflow which documented the process of correcting data discrepancies identified through quality syntaxes. For each problem identified, a query was opened and directed to the user who performed the data collection. The responses were subsequently reviewed by the study coordinator who was responsible for finalizing this data quality assessment process. More than 150 queries were resolved in the Prospective Multicenter CIED Registry.
