# Supplementary material for: The impact of data quality monitoring of a multicenter prospective registry of cardiac implantable electronic devices
Source: MethodsX. 2023 Oct 20;11:102454. doi: 10.1016/j.mex.2023.102454 (PMC10618759; doi:10.1016/j.mex.2023.102454)
Supplement: Supplementary file 3 [file mmc3.pdf]

Table S3 - Overall rate of data quality metrics of the Multicenter Prospective CIED Registry according to research site.

Research Site	Data collection method	n (%)	Completeness mean (95% CI)	Accuracy mean (95% CI)	Temporal plausibility mean (95% CI)
Coordinating center	Online, off-line, surveys	1,982 (75.3)	99.9 (99.9 - 100.0)	99.9 (99.9 - 100.0)	99.3 (98.8 - 99.8)
Site 1	Online	255 (9.7)	99.9 (99.8 - 100.0)	99.4 (98.5 - 100.0)	88.5 (86.7 - 90.4)
Site 2	Online	81 (3.1)	99.5 (98.5 - 100.0)	98.8 (97.1 - 100.0)	92.3 (89.1 - 95.6)
Site 3	Online	181 (6.9)	99.2 (98.2 - 100.0)	99.2 (98.2 - 100.0)	98.3 (98.1 - 98.5)
Site 4	Online	132 (5.0)	99.6 (98.7 - 100.0)	98.9 (97.8 - 100.0)	45.8 (37.8 - 53.8)
